# Supplementary material for: Tracking Conditioned Fear in Pair-Housed Mice Using Deep Learning and Real-Time Cue Delivery
Source: bioRxiv. 2025 May 15:2025.05.10.653260. Preprint. [Version 1] doi: 10.1101/2025.05.10.653260 (PMC12132537; doi:10.1101/2025.05.10.653260)
Supplement: Supplement 3 — S2. Table 2: Timeline of behavioral testing and pseudorandom home cage CS exposure. Mice were habituated in the home cage room before undergoing a two-day shock box habituation period (20 min and 40 min, respectively) followed by fear conditioning (Day 1). Conditioned stimulus (CS) presentations were delivered in the home cage in a pseudorandomized on/off schedule across 7 test days (Days 3–24). Each test day consisted of a total of six CS trials at different timepoints, 3 CS across the light phase and 3 CS across the dark. Subsequent behavioral assays included fear expression testing in a novel shock box context (Day 24), Elevated Plus Maze (EPM, Day 25), pre-pulse inhibition (PPI, Day 26), and fear-potentiated startle (FPS, Day 27). [file media-3.pdf]

Supplemental 2: Table 2

Timeline	Location	Days	Pseudorandom CS and Behavioral Test
	Home Cage Room	Habituation	Placed in cages 2pm
	Shock Box	20m Habituation	11am
	Shock Box	40m Habituation	10am
1	Shock Box	Fear Conditioning	12pm
3	Homecage	Homecage CS Day 1	10:10, 13:52*, 17:34, 19:25, 2:49, 6:31
5	Homecage	Homecage CS Day 2	10:22*, 17:46, 19:37, 4:52, 6:43, 8:34
7	Homecage	Homecage CS Day 3	10:18*, 17:42, 21:24, 2:57. 4:48, 8:30
9	Homecage	Homecage CS Day 4	12:12*, 18:04, 19:32, 21:00, 1:24, 11:40
11	Homecage	Homecage CS Day 5	9:58*, 18:10, 0:19, 4:25, 6:28, 8:31
14	Homecage	Homecage CS Day 6	10:56*, 13:44, 16:32, 22:08, 13:32, 5:08
16	Homecage	Homecage CS Day 7	12:22*, 17:58, 23:34, 3:46, 6:34, 10:46
24	Shock Box – novel context	Fear expression (2CS)	12pm
25	Behavior room	EPM	11am
26	Startle Box	PPI	10am
27	Startle Box	FPS	10am
